# GDPR-anonymous population overlap estimation

**DOI:** 10.23889/ijpds.v9i5.2712

**Published:** 2024-09-18

**Authors:** Patrick Bowman, Carol Flannagan, Andrew Leslie, Helen Spradlin

**Affiliations:** 1 University of Michigan Transportation Research Institute

In Michigan, police crash reports are the primary source of data on traffic crashes. Though many aspects are very reliable, some details are difficult to observe or require subjective judgement. Injury severity, which is central to the road safety issue, unfortunately falls into the second category. The KABCO injury scale used by law enforcement classifies injuries as “possible,” “suspected minor,” and “suspected serious” which may not correspond to determinations made by medical professionals. EMS records can provide a means to improve crash injury data using medical evaluations, but these records are not linked to the police reports.

This project explores the linkage of police crash reports and EMS records using both Michigan data sources from 2018 to 2021. Given the association between severity of injuries and person type, three crash subsets with high injury risk were the focus: pedestrians, bicyclists, and motorcyclists. Collectively, these person types represent 25.1% of the police reported fatalities and suspected serious injuries in Michigan in 2022 despite being only 1.2% of the crashing population.

Person-level records for pedestrians, bicyclists, and motorcyclists were identified and divided into three separate datasets for each data source. EMS data events were restricted cases believed to be associated with traffic crashes using ICD-10 injury codes. Personal identifiers were not provided, so probabilistic linkage was used to link person crash records to EMS records. Event date, location, ambulance ID, and person demographic variables were utilized. Linkage rates and injury severity agreement was computed to assess differences between the two datasets.

